# Supplementary material for: Integration of machine learning and genome-scale metabolic modeling identifies multi-omics biomarkers for radiation resistance
Source: Nat Commun. 2021 May 11;12:2700. doi: 10.1038/s41467-021-22989-1 (PMC8113601; doi:10.1038/s41467-021-22989-1)
Supplement: Supplementary file 1 — Supplementary Information [file 41467_2021_22989_MOESM1_ESM.pdf]

Supplementary Figures:

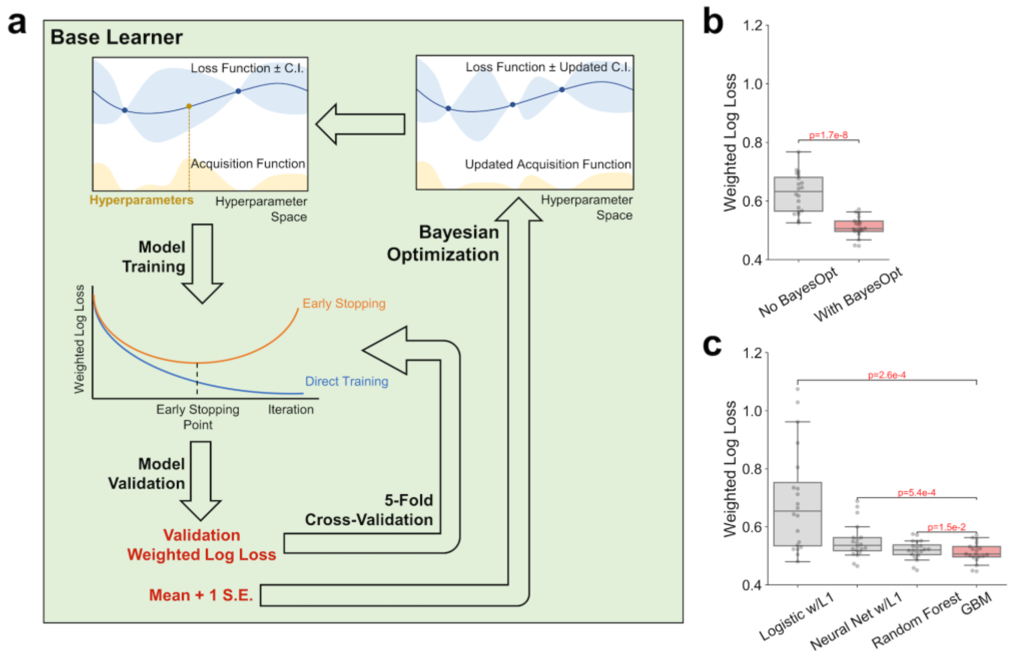

Supplementary Fig. 1 | Base learner used in the gene expression, multi-omics, and non-invasive classifiers for radiation response. a, Base learner performing two-class classification of radiation response, utilizing a gradient boosting machine (GBM) algorithm with Bayesian optimization, early stopping, and 5-fold cross validation to determine optimal hyperparameter values. Note that for the gene expression classifier, this base learner is not integrated with other base learners or a meta-learner as only one dataset is utilized. **b-c,** Performance of classifier trained on gene expression data from TCGA tumors, comparing (**b**) use of Bayesian optimization compared to no Bayesian optimization, and (**c**) GBM-based classifiers versus other machine learning algorithms. n = 20 training+validation/testing splits. Boxplots: box = 25th, 50th, and 75th percentiles, whiskers = 1.5 times the interquartile range. Statistical test: two-sided t-test.

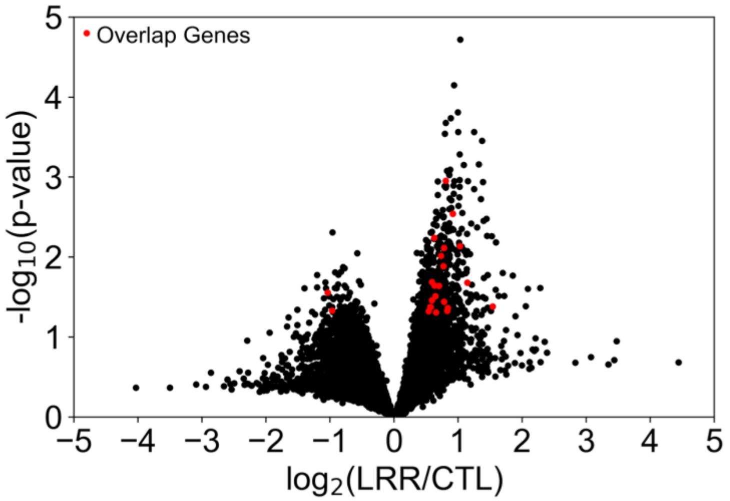

Supplementary Fig. 2 | Differential gene expression between locoregional recurrence (LRR) and non-recurrent control (CTL) breast cancer tumors from the Keene et al. dataset. Statistically significant genes that were also among the 782 significant genes from our gene expression classifier trained on TCGA data are highlighted in red and listed in **Supplementary Table 2**.

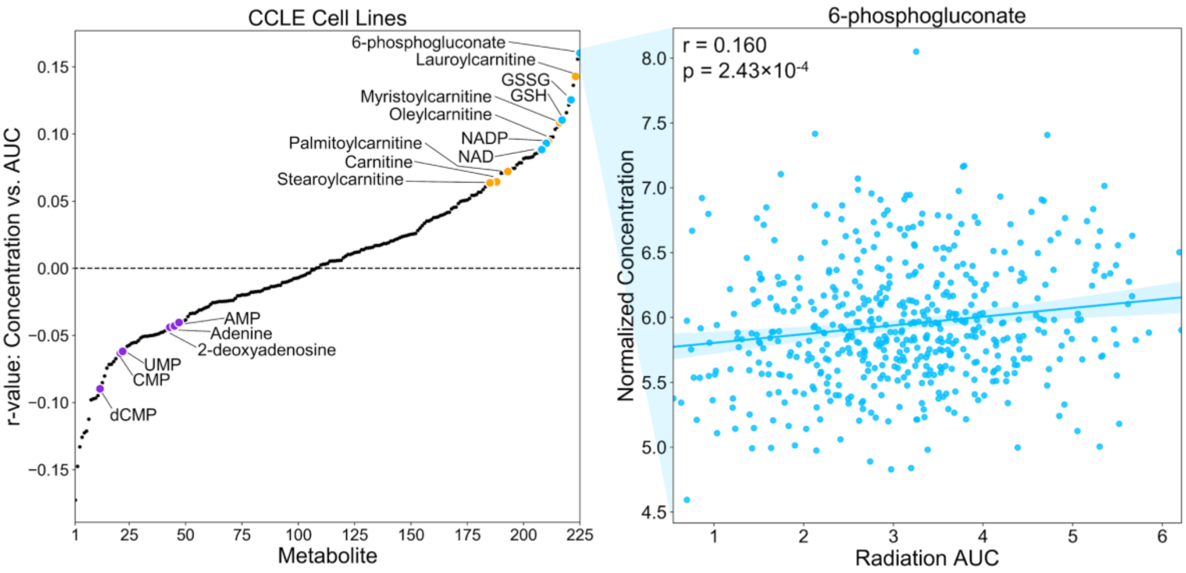

Supplementary Fig. 3 | Association between cellular metabolomics and radiation response among the CCLE panel of cancer cell lines. (Left) Correlation between metabolite concentration and radiation resistance (measured using AUC metric) among 225 experimentally-measured metabolites in the CCLE panel. Metabolite classes are colored as in Figure 2. (Right) Example regression between metabolite concentration and AUC for 6-phosphogluconate. Statistical test: one-sample correlation t-test.

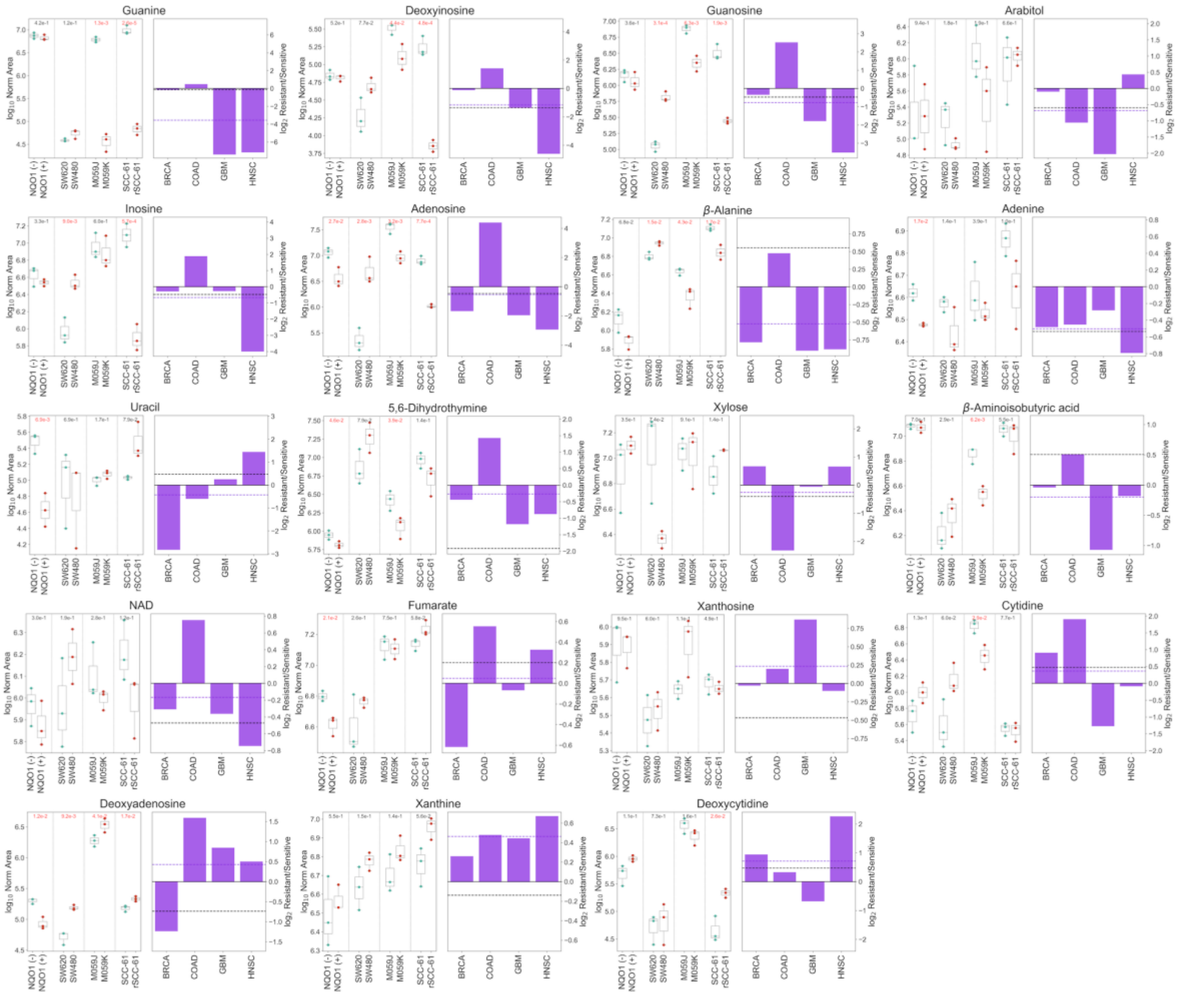

Supplementary Fig. 4 | Experimentally-measured concentrations of nucleotide metabolites in matched radiation-sensitive and -resistant cell lines. (Left) Replicate metabolite concentrations from all four cell line pairs, with values expressed as the $\log_{10}$ normalized area from LC-MS/MS. (Right) Bars: Ratio value for each cell line pair, expressed as the $\log_2$ ratio of mean radiation-resistant concentration versus mean radiation-sensitive concentration. Colored line: mean experimental $\log_2$ Resistant/Sensitive across all four cell line pairs. Black line: FBA model-predicted $\log_2$ ratio of average metabolite production in radiation-resistant versus -sensitive TCGA tumors. n = 3 replicates. Boxplots: box = 25th, 50th, and 75th percentiles, whiskers = 1.5 times the interquartile range. Statistical test: two-sided t-test.

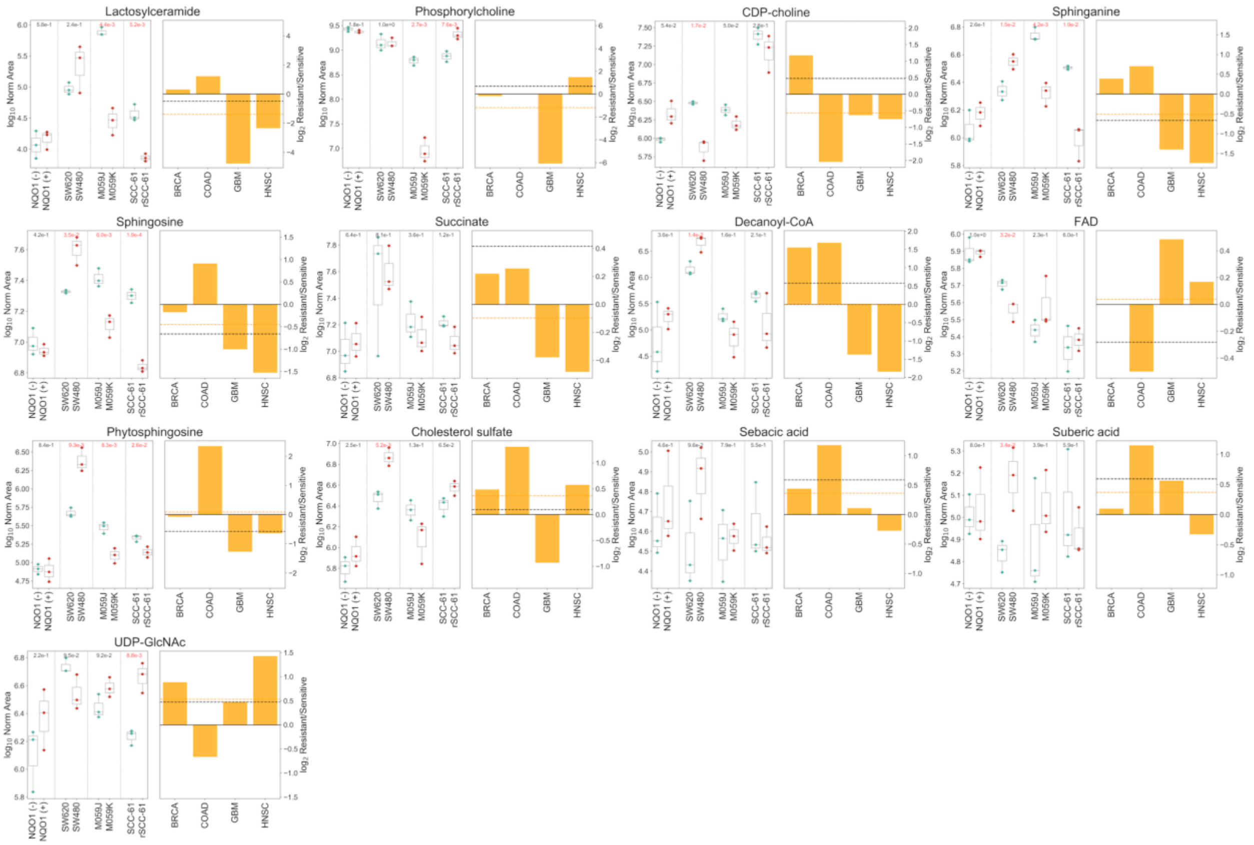

Supplementary Fig. 5 | Experimentally-measured concentrations of lipid metabolites in matched radiation-sensitive and -resistant cell lines. (Left) Replicate metabolite concentrations from all four cell line pairs, with values expressed as the $\log_{10}$ normalized area from LC-MS/MS. (Right) Bars: Ratio value for each cell line pair, expressed as the $\log_2$ ratio of mean radiation-resistant concentration versus mean radiation-sensitive concentration. Colored line: mean experimental $\log_2$ Resistant/Sensitive across all four cell line pairs. Black line: FBA model-predicted $\log_2$ ratio of average metabolite production in radiation-resistant versus -sensitive TCGA tumors. n = 3 replicates. Boxplots: box = 25th, 50th, and 75th percentiles, whiskers = 1.5 times the interquartile range. Statistical test: two-sided t-test.

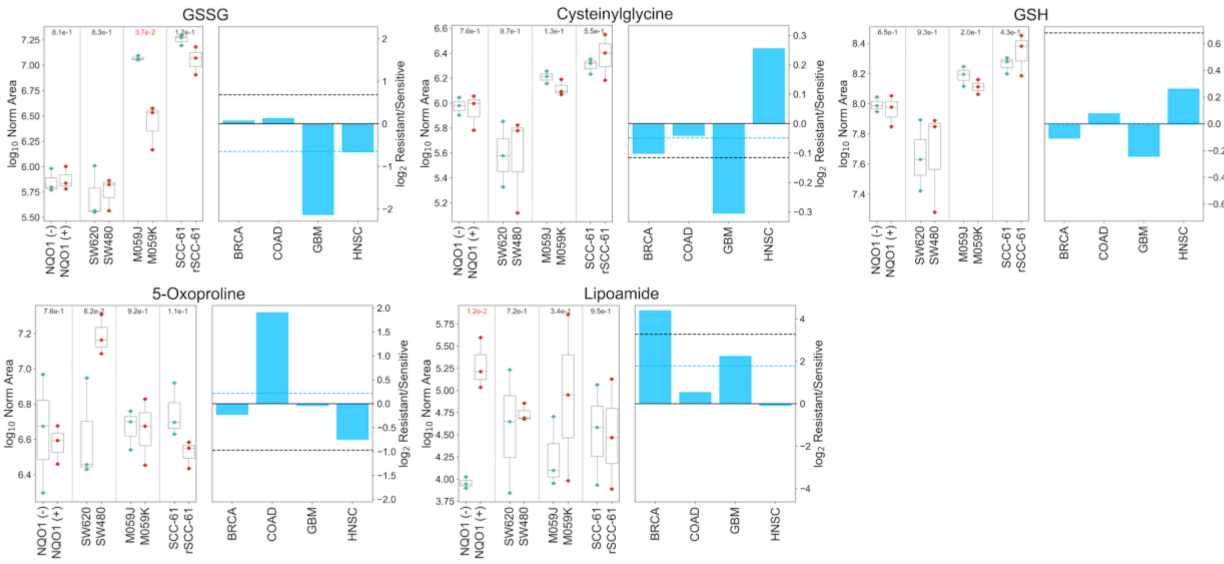

Supplementary Fig. 6 | Experimentally-measured concentrations of cysteine/antioxidant metabolites in matched radiation-sensitive and -resistant cell lines. (Left) Replicate metabolite concentrations from all four cell line pairs, with values expressed as the $\log_{10}$ normalized area from LC-MS/MS. (Right) Bars: Ratio value for each cell line pair, expressed as the $\log_2$ ratio of mean radiation-resistant concentration versus mean radiation-sensitive concentration. Colored line: mean experimental $\log_2$ Resistant/Sensitive across all four cell line pairs. Black line: FBA model-predicted $\log_2$ ratio of average metabolite production in radiation-resistant versus -sensitive TCGA tumors. n = 3 replicates. Boxplots: box = 25th, 50th, and 75th percentiles, whiskers = 1.5 times the interquartile range. Statistical test: two-sided t-test.

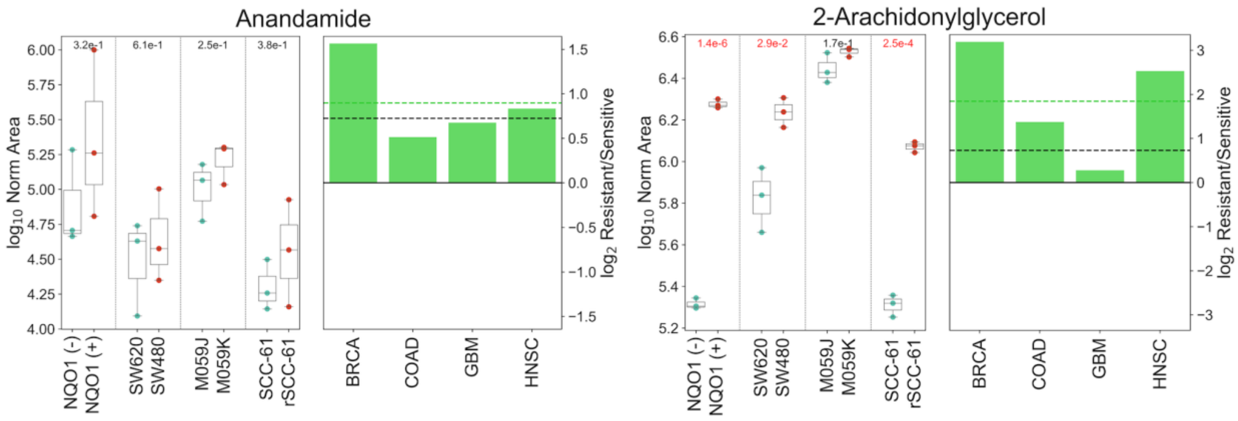

Supplementary Fig. 7 | Experimentally-measured concentrations of immune system mediating metabolites in matched radiation-sensitive and -resistant cell lines. (Left) Replicate metabolite concentrations from all four cell line pairs, with values expressed as the $\log_{10}$ normalized area from LC-MS/MS. (Right) Bars: Ratio value for each cell line pair, expressed as the $\log_2$ ratio of mean radiation-resistant concentration versus mean radiation-sensitive concentration. Colored line: mean experimental $\log_2$ Resistant/Sensitive across all four cell line pairs. Black line: FBA model-predicted $\log_2$ ratio of average metabolite production in radiation-resistant versus -sensitive TCGA tumors. n = 3 replicates. Boxplots: box = 25th, 50th, and 75th percentiles, whiskers = 1.5 times the interquartile range. Statistical test: two-sided t-test.

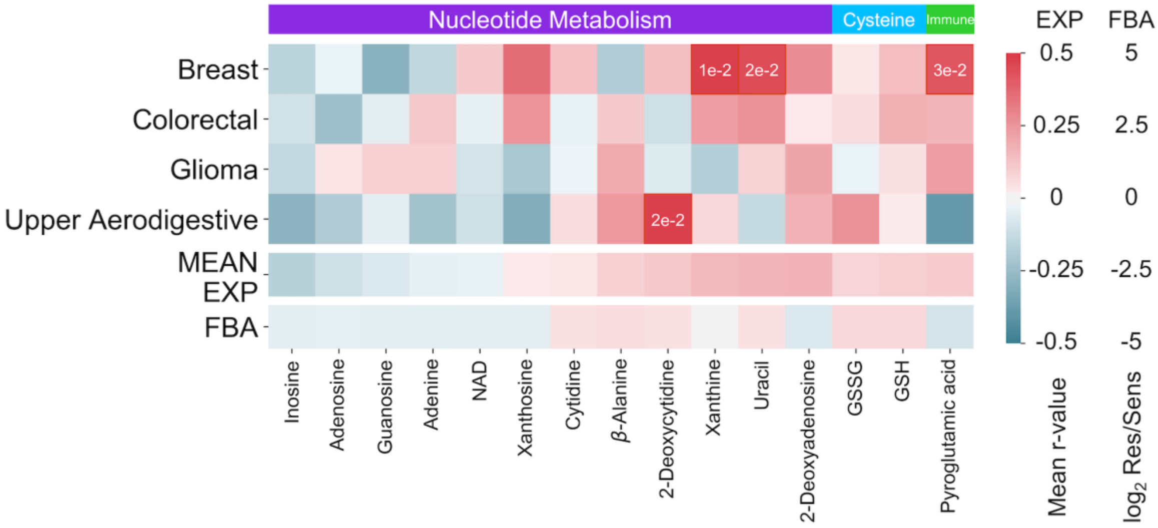

Supplementary Fig. 8 | Comparison of FBA model-predicted differential metabolite production in TCGA tumors with experimental correlations with radiation AUC in CCLE cell lines among 4 cancer types. Breast, Colorectal, Glioma, Upper Aerodigestive: average Pearson correlation coefficient between metabolite concentration and cell line AUC among the cell lines listed in **Supplementary Table 4**. Statistically-significant correlations within each cancer type are represented by box outlines and p-values. Number of cell lines for each cancer type is provided in **Supplementary Table 4**. Statistical test: two-sided t-test. MEAN EXP: averaged experimental value across all four cancer types. FBA: $\log_2$ ratio of FBA model-predicted metabolite production rates in radiation-resistant versus -sensitive TCGA tumors.

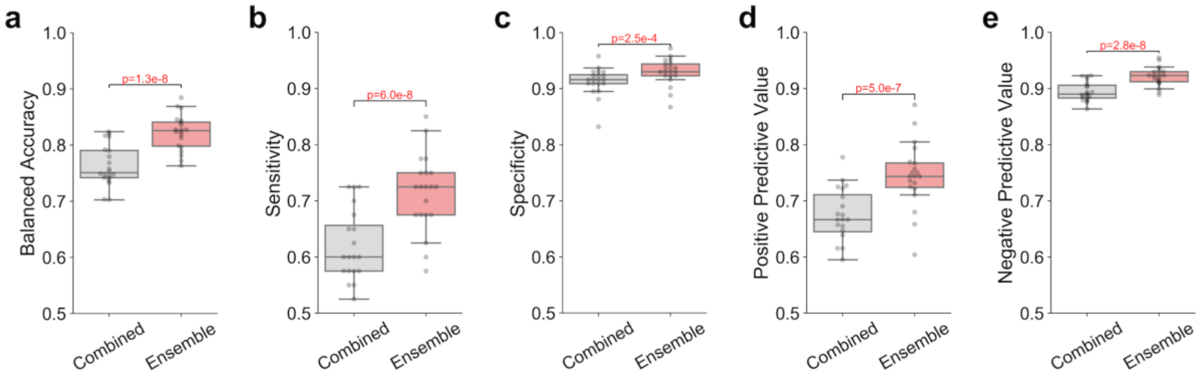

Supplementary Fig. 9 | Performance of the multi-omics classifier, comparing the dataset-independent ensemble architecture versus combining datasets together before training on a single classifier. Multiple alternative classifier performance metrics are provided. n = 20 training+validation/testing splits. Boxplots: box = 25th, 50th, and 75th percentiles, whiskers = 1.5 times the interquartile range. Statistical test: two-sided t-test.

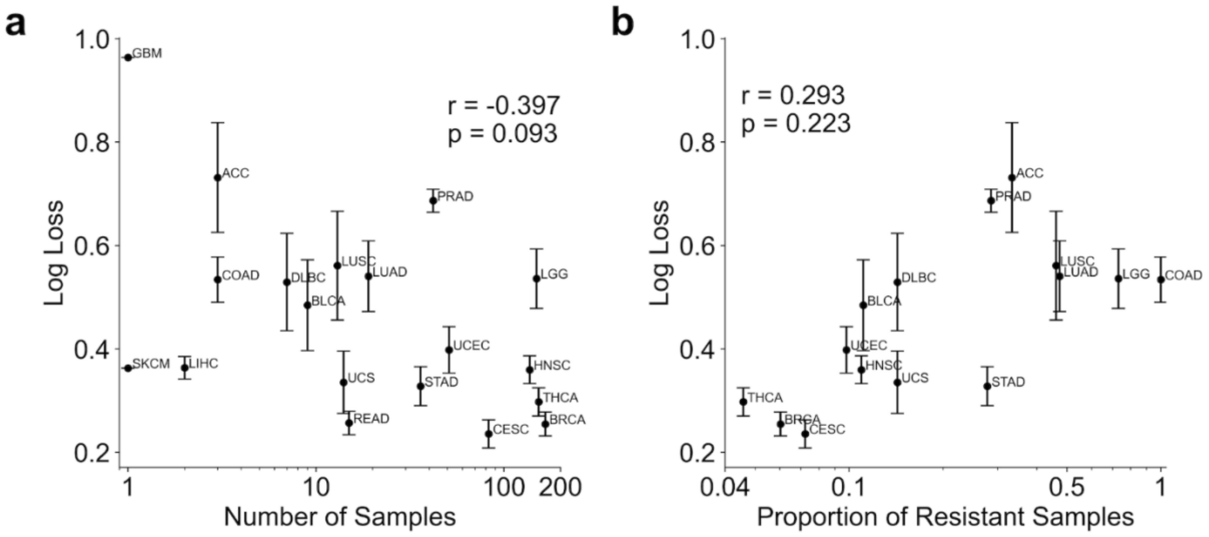

Supplementary Fig. 10 | Comparison of multi-omics classifier performance on samples from different cancer types. a, Correlation between sample log loss and number of samples within each cancer type. Error bars: mean ± 1 standard error. Statistical test: one-sample correlation t-test. **b,** Correlation between sample log loss and proportion of radiation-resistant samples within each cancer type. Number of samples in each cancer types is provided in Dataset 1. Error bars: mean ± 1 standard error. Statistical test: one-sample correlation t-test.

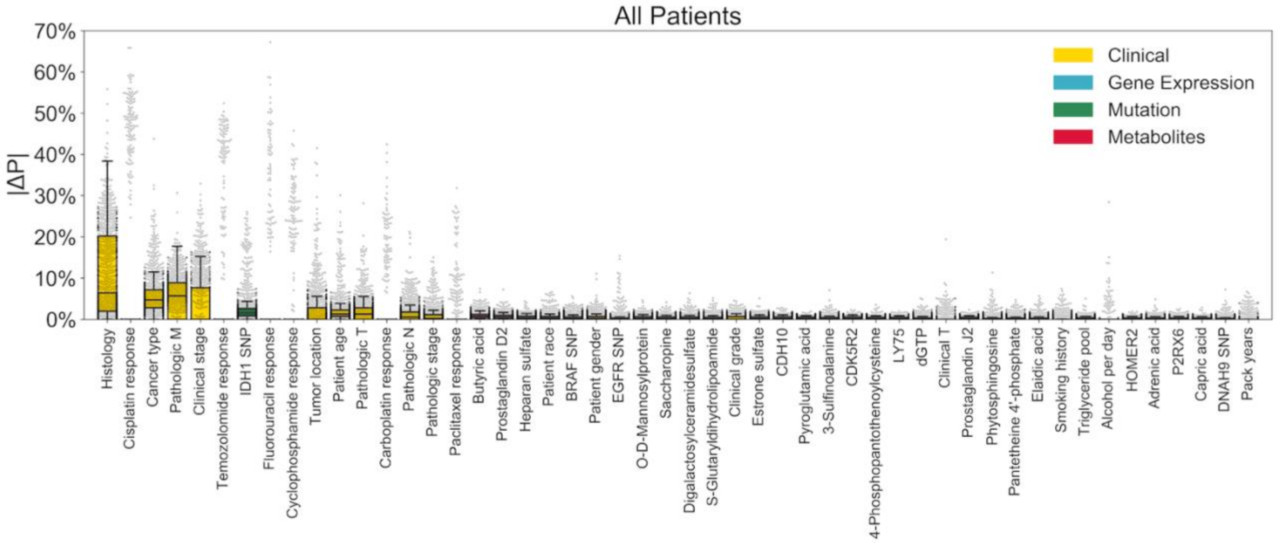

Supplementary Fig. 11 | Top 50 features with largest mean |ΔP| scores, showing individual sample values. n = 904 samples. Boxplots: box = 25th, 50th, and 75th percentiles, whiskers = 1.5 times the interquartile range.

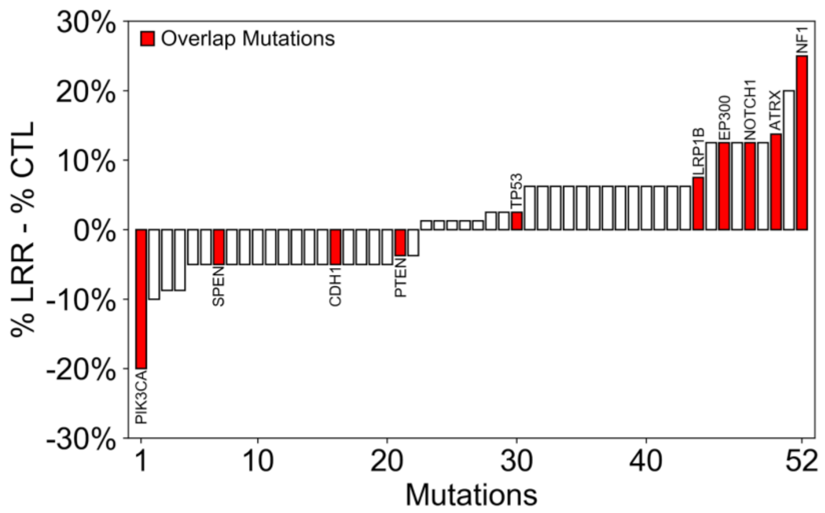

Supplementary Fig. 12 | Differences in gene mutation rates between locoregional recurrence (LRR) and non-recurrent control (CTL) breast cancer tumors from the Keene et al. dataset. Gene mutations that were among the 105 gene mutations within the 725 significant features from our multi-omics classifier trained on TCGA data are highlighted in red with their gene names given.

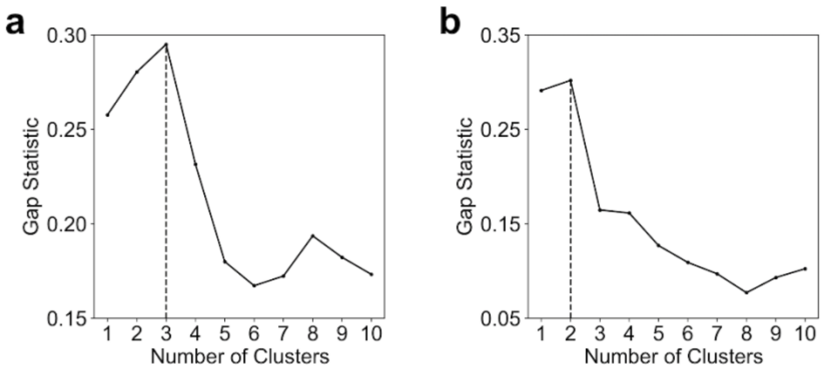

Supplementary Fig. 13 | *k*-Means clustering of clinical dataset contributions for individual samples. Gap statistic values for each value of *k* are shown for the (**a**) multi-omics classifier, and (**b**) non-invasive classifier.

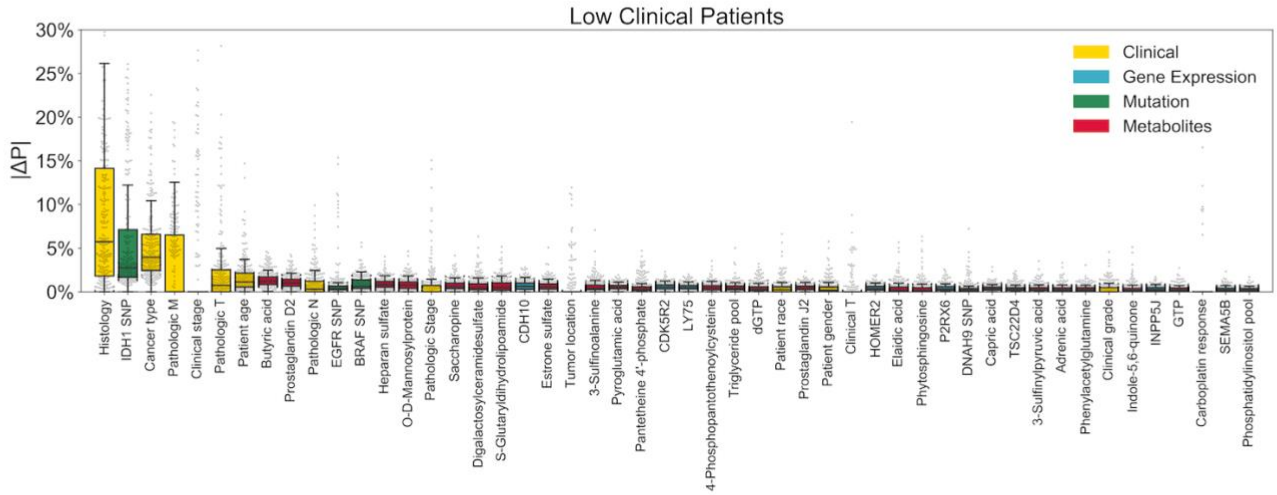

Supplementary Fig. 14 | Top 50 features with largest mean |ΔP| scores among samples within the “Low Clinical” cluster, showing individual sample values. n = 249 samples. Boxplots: box = 25th, 50th, and 75th percentiles, whiskers = 1.5 times the interquartile range.

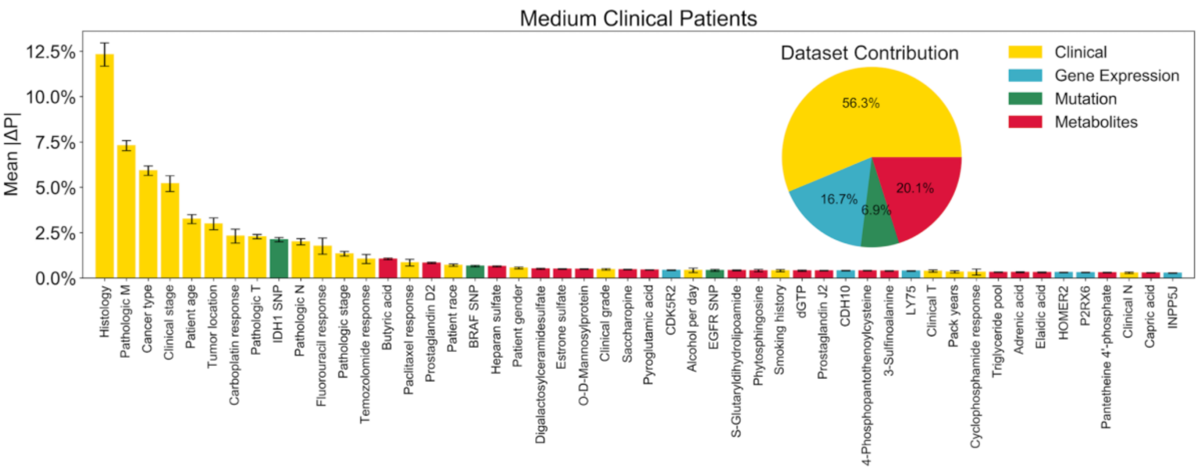

Supplementary Fig. 15 | Top 50 features with largest mean |ΔP| scores among samples within the "Medium Clinical" cluster. (Inset) Relative contribution of features from each dataset to the sum of total mean |ΔP| scores, averaged across all samples within the "Medium Clinical" cluster. n = 324 samples. Error bars: mean ± 1 standard error.

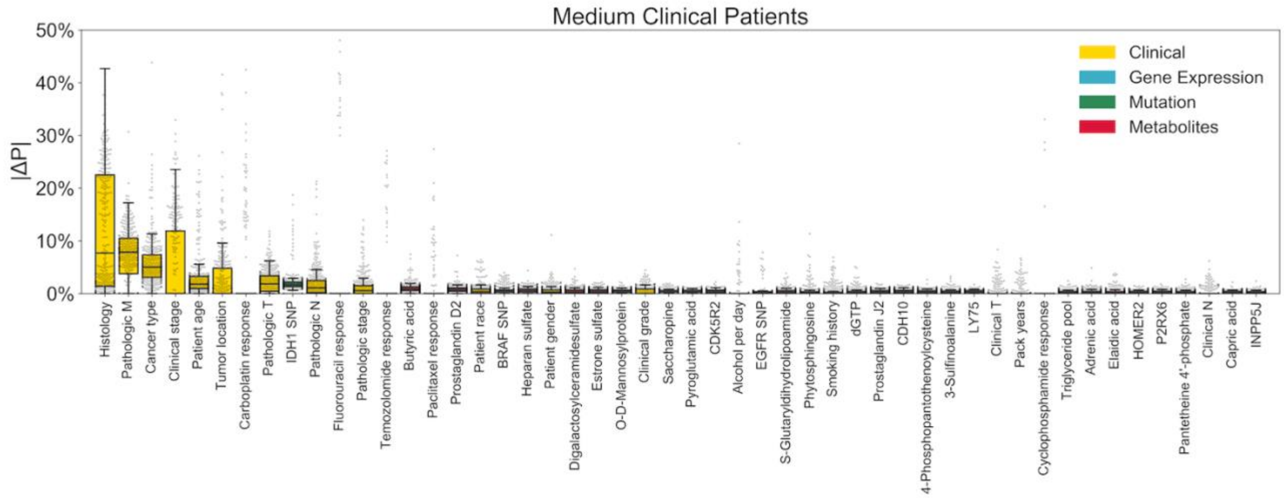

Supplementary Fig. 16 | Top 50 features with largest mean |ΔP| scores among samples within the "Medium Clinical" cluster, showing individual sample values. n = 324 samples. Boxplots: box = 25th, 50th, and 75th percentiles, whiskers = 1.5 times the interquartile range.

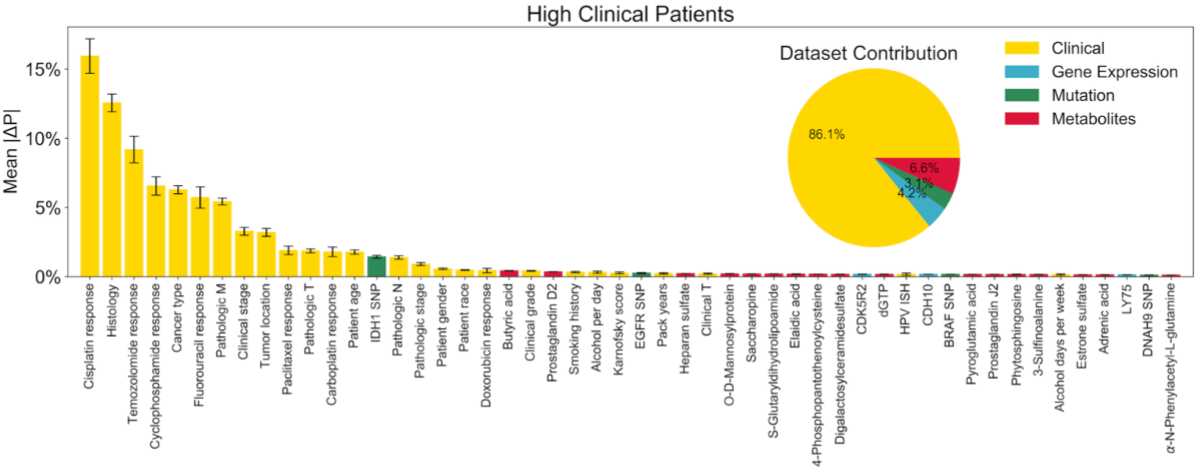

Supplementary Fig. 17 | Top 50 features with largest mean |ΔP| scores among samples within the "High Clinical" cluster. (Inset) Relative contribution of features from each dataset to the sum of total mean |ΔP| scores, averaged across all samples within the "High Clinical" cluster. n = 331 samples. Error bars: mean ± 1 standard error.

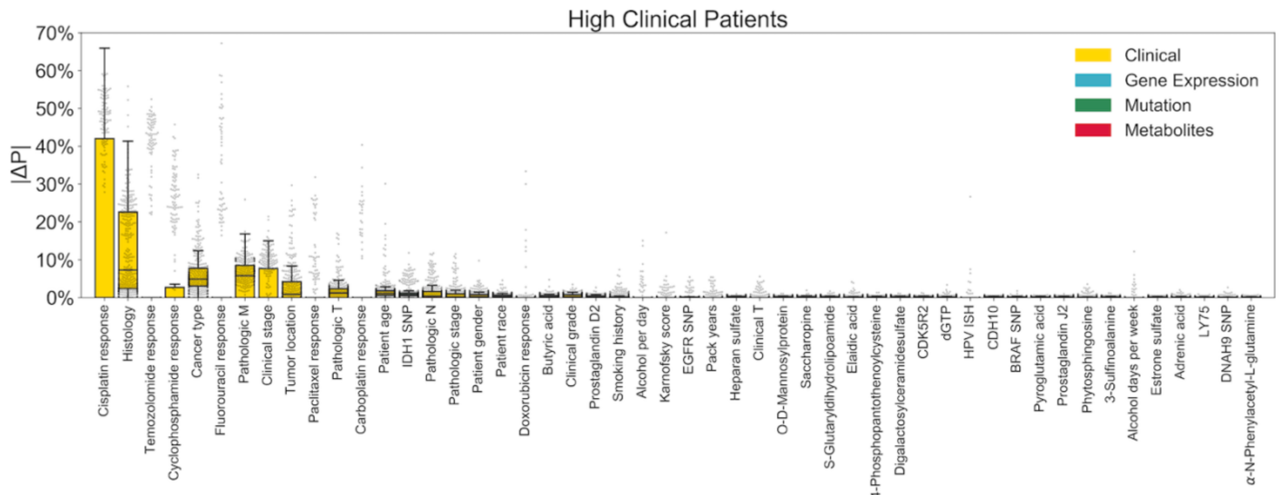

Supplementary Fig. 18 | Top 50 features with largest mean |ΔP| scores among samples within the "High Clinical" cluster, showing individual sample values. n = 331 samples. Boxplots: box = 25th, 50th, and 75th percentiles, whiskers = 1.5 times the interquartile range.

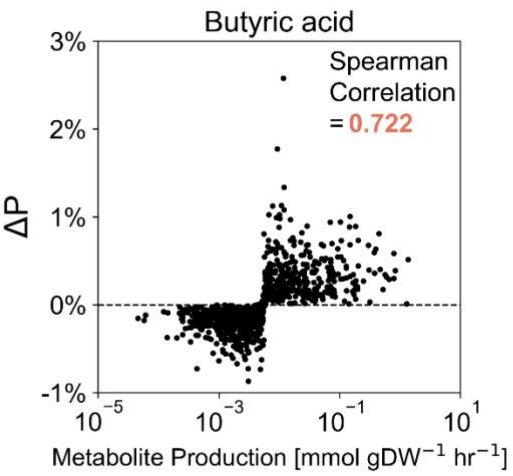

Supplementary Fig. 19 | Regression between SHAP values and predicted metabolite production rate for a representative metabolite. Values are shown for each individual patient tumor.

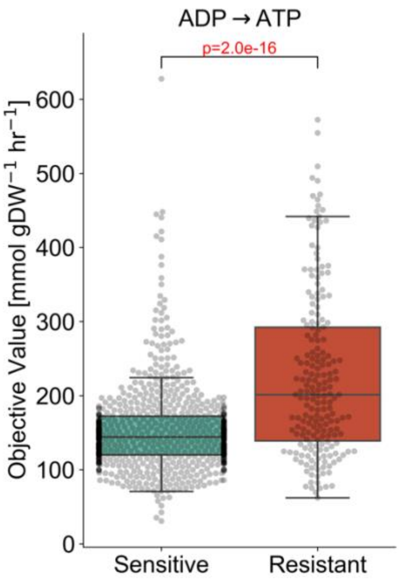

Supplementary Fig. 20 | Comparison of FBA model-predicted maximal conversion of ADP to ATP between radiation-sensitive and -resistant TCGA tumors. n = 716 radiation-sensitive samples, 199 radiation-resistant samples. Boxplots: box = 25th, 50th, and 75th percentiles, whiskers = 1.5 times the interquartile range. Statistical test: two-sided t-test.

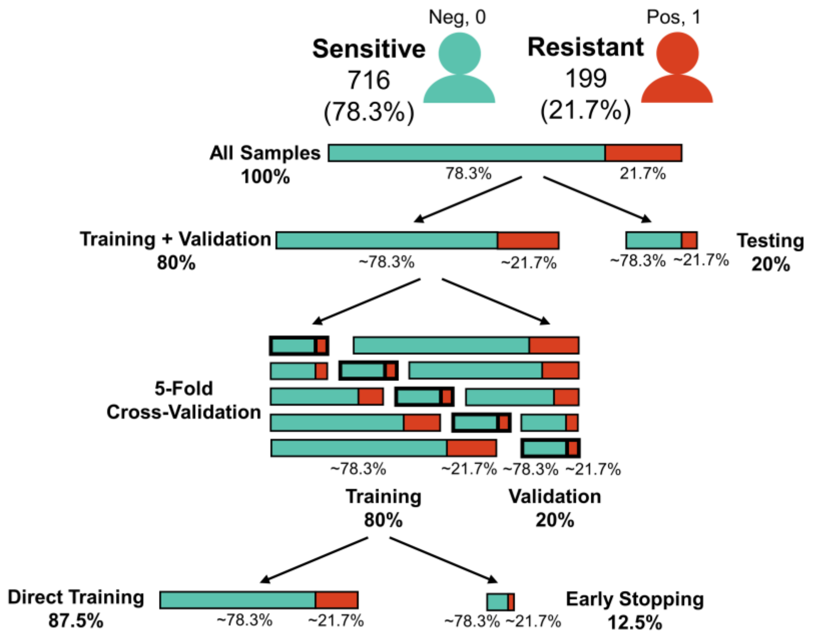

Supplementary Fig. 21 | Data splitting for classifier training and testing.

Supplementary Tables:

Supplementary Table 1 | Overlapping genes among those with statistically-significant differential gene expression in the Keene et al. dataset and those among the 782 significant genes from our gene expression classifier trained on TCGA data.

ACER3	DDR1	FLNB	ITPK1	NAV2	RPN1
CDH1	DGAT2	GABRB3	KRT18	PRUNE2	SEMA3F
CHD7	ELFN2	HSP90B1	KRT23	RCC2	SPON1
CHMP4C	ENTHD1	ILDR2	LRRC8A	RDX	

Supplementary Table 2 | Matched radiation-sensitive and radiation-resistant cancer cell lines

Cancer Type	Radiation-Sensitive	Radiation-Resistant	Notes	Source
Breast (BRCA)	MDA-MB-231 NQO1 (-)	MDA-MB-231 NQO1 (+)	Stable NQO1 expression was restored in NQO1(-) cells to create NQO1(+) cells.	Dr. David Boothman, Indiana University[91]
Colon (COAD)	SW620	SW480	Primary tumor (SW480) and lymph node metastasis (SW620) from the same patient, with different radiation sensitivities.	ATCC
Glioblastoma (GBM)	M059J	M059K	Both isolated from same tumor specimen. M059J cells lack DNA-PK activity, rendering them more radiation-sensitive.	ATCC
Head and Neck (HNSC)	SCC-61	rSCC-61	rSCC-61 cells were derived from SCC-61 cells after repeated radiation exposure and selection of surviving colonies.	Dr. Cristina Furdui, Wake Forest University[92,93]

Supplementary Table 3 | Hyperparameter ranges for Bayesian optimization with gradient boosting machine classifiers.

Parameter Name	Distribution	Lower Bound	Upper Bound
eta	log-uniform	0.01	0.5
gamma	log-uniform	0	5
max_depth	uniform	1	11
subsample	uniform	0.5	1
colsample_bytree	uniform	0.5	1
colsample_bylevel	uniform	0.5	1
reg_lambda	log-uniform	1	4
reg_alpha	log-uniform	0	1

Supplementary Table 4 | Hyperparameter ranges for Bayesian optimization with the random forest classifier.

Parameter Name	Distribution	Lower Bound	Upper Bound
n_estimators	log-uniform	1	1000
criterion	choice	entropy, gini	
max_depth	uniform	1	11
min_samples_split	uniform	0	1
min_samples_leaf	uniform	0	0.5
max_features	choice	log2, sqrt	

Supplementary Table 5 | Hyperparameter ranges for Bayesian optimization with the logistic regression classifier with L1 regularization.

Parameter Name	Distribution	Lower Bound	Upper Bound
C	log-uniform	0.001	1000

Supplementary Table 6 | Hyperparameter ranges for Bayesian optimization with the neural network classifier with L1 regularization.

Parameter Name	Distribution	Lower Bound	Upper Bound
Number of layers	uniform	1	5
Neurons per layer	choice	8, 16, 32, 64, 128, 256, 512, 1024	
activation_function	choice	elu, relu, sigmoid	
optimizer	choice	adam, rmsprop, sgd	
l1	log-uniform	0.000001	0.1
dropout	uniform	0	0.5
