# Supplementary material for: Integration of machine learning and genome-scale metabolic modeling identifies multi-omics biomarkers for radiation resistance
Source: Nat Commun. 2021 May 11;12:2700. doi: 10.1038/s41467-021-22989-1 (PMC8113601; doi:10.1038/s41467-021-22989-1)
Supplement: Supplementary file 2 — Description of Additional Supplementary Files [file 41467_2021_22989_MOESM2_ESM.pdf]

Description of Additional Supplementary Files

File Name: Supplementary Data 1
Description: All supplementary data referenced within the main document. These files are also on Github including significant genes, mean SHAP values
