# Supplementary material for: Integration of machine learning and genome-scale metabolic modeling identifies multi-omics biomarkers for radiation resistance
Source: Nat Commun. 2021 May 11;12:2700. doi: 10.1038/s41467-021-22989-1 (PMC8113601; doi:10.1038/s41467-021-22989-1)
Supplement: Supplementary file 4 — Reporting Summary [file 41467_2021_22989_MOESM4_ESM.pdf]

nature research

Corresponding author(s): Melissa L. Kemp

Last updated by author(s): Feb 17, 2021

Reporting Summary

Nature Research wishes to improve the reproducibility of the work that we publish. This form provides structure for consistency and transparency in reporting. For further information on Nature Research policies, see our Editorial Policies and the Editorial Policy Checklist.

Statistics

For all statistical analyses, confirm that the following items are present in the figure legend, table legend, main text, or Methods section.

n/a	Confirmed	
[ ]	[x]	The exact sample size (*n*) for each experimental group/condition, given as a discrete number and unit of measurement
[ ]	[x]	A statement on whether measurements were taken from distinct samples or whether the same sample was measured repeatedly
[ ]	[x]	The statistical test(s) used AND whether they are one- or two-sided<br>*Only common tests should be described solely by name; describe more complex techniques in the Methods section.*
[ ]	[x]	A description of all covariates tested
[ ]	[x]	A description of any assumptions or corrections, such as tests of normality and adjustment for multiple comparisons
[ ]	[x]	A full description of the statistical parameters including central tendency (e.g. means) or other basic estimates (e.g. regression coefficient) AND variation (e.g. standard deviation) or associated estimates of uncertainty (e.g. confidence intervals)
[ ]	[x]	For null hypothesis testing, the test statistic (e.g. *F*, *t*, *r*) with confidence intervals, effect sizes, degrees of freedom and *P* value noted<br>*Give P values as exact values whenever suitable.*
[x]	[ ]	For Bayesian analysis, information on the choice of priors and Markov chain Monte Carlo settings
[x]	[ ]	For hierarchical and complex designs, identification of the appropriate level for tests and full reporting of outcomes
[ ]	[x]	Estimates of effect sizes (e.g. Cohen's *d*, Pearson's *r*), indicating how they were calculated

Our web collection on statistics for biologists contains articles on many of the points above.

Software and code

Policy information about availability of computer code

Data collection	Metabolomics data: Compound Discoverer 3.1
Data analysis	Machine learning classifiers: Hyperopt (v0.2.3), Keras (v2.3.1), NumPy (v1.18.4), Pandas (v1.0.3), scikit-learn (v0.21.2), SciPy (v1.4.1), TensorFlow(v2.2.0), XGBoost (v0.90), custom Python code (provided at https://github.com/ kemplab/ML-radiation)<br>Data analysis: Matplotlib(v3.1.0), SHAP (v0.37.0), Seaborn (v0.10.1), custom Python code (provided at https://github.com/kemplab/ML-radiation)

For manuscripts utilizing custom algorithms or software that are central to the research but not yet described in published literature, software must be made available to editors and reviewers. We strongly encourage code deposition in a community repository (e.g. GitHub). See the Nature Research guidelines for submitting code & software for further information.

Data

Policy information about availability of data

All manuscripts must include a data availability statement. This statement should provide the following information, where applicable:

- Accession codes, unique identifiers, or web links for publicly available datasets
- A list of figures that have associated raw data
- A description of any restrictions on data availability

Databases: TCGA (GDC Portal - https://portal.gdc.cancer.gov; Synapse TCGA___Pancancer project - https://www.synapse.org/#!Synapse:syn300013/wiki/70804; Rahman et al. – GEO: GSE62944), NCI-60 (NCI DTP – https://wiki.nci.nih.gov/display/NCIDTPdata/Molecular+Target+Data), CCLE (Broad Institute – https://data.broadinstitute.org/ccle/CCLE_RNAseq_rsem_genes_tpm_20180929.txt.gz), Keene et al. (GEO: GSE119937), KEGG (https://genome.jp). The following datasets are available at https://github.com/kemplab/ML-radiation:

- Dataset 1. TCGA samples included in the analysis, with corresponding radiation response and patient/tumor factors
- Dataset 2. SHAP values (AP) from the gene expression classifier, for individual TCGA patients

- ~~Dataset 3. Mean absolute SHAP values (mean |ΔP|) for individual features from the gene expression classifier~~
- ~~Dataset 4. FBA model-predicted metabolite production rates in TCGA tumors~~
- ~~Dataset 5. Experimental metabolomics data from radiation-sensitive and -resistant cancer cell lines~~
- ~~Dataset 6. Comparison of model-predicted and experimentally-validated metabolite levels in radiation-sensitive and -resistant cancers~~
- ~~Dataset 7. SHAP values (ΔP) from the multi-omics classifier, for individual TCGA patients~~
- ~~Dataset 8. Mean absolute SHAP values (mean |ΔP|) for individual features from the multi-omics classifier~~
- ~~Dataset 9. SHAP values (ΔP) from the non-invasive classifier, for individual TCGA patients~~
- ~~Dataset 10. Mean absolute SHAP values (mean |ΔP|) for individual features from the non-invasive classifier~~
- ~~Dataset 11. Frequency of SNPs within each gene among all 915 TCGA samples from this study~~

Jupyter notebooks and datasets related to the generation of personalized genome-scale FBA models of TCGA tumors are available at https://github.com/kemplab/FBA-pipeline.

Field-specific reporting

Please select the one below that is the best fit for your research. If you are not sure, read the appropriate sections before making your selection.

[x] Life sciences [ ] Behavioural & social sciences [ ] Ecological, evolutionary & environmental sciences

For a reference copy of the document with all sections, see nature.com/documents/nr-reporting-summary-flat.pdf

Life sciences study design

All studies must disclose on these points even when the disclosure is negative.

Sample size	Samples sizes for computational analysis were decided based upon the availability of samples (Number of TCGA/NCI-60/CCLE/Keene et al. patient samples with available radiation sensitivity and transcriptomic data). Sample sizes for experimental metabolomics on cancer cell line pairs were determined based on the availability of metabolomics resources. The sufficiency of these sample sizes is evident by the statistical significance of statistical tests on the results from these samples.
Data exclusions	Samples from TCGA/NCI-60/CCLE/Keene et al. datasets were excluded if either radiation sensitivity or transcriptomic data were not available for these samples. Exclusion data were pre-established.
[illegible]eplication	Reproducibility of experimental metabolomics findings was verified through comparing findings between three biological replicates for each cell line.
Randomization	Samples were allocated into groups based on radiation sensitivity. All covariates were used as features in the machine learning classifiers.
[illegible]inding	Blinding was performed during execution of the experimental metabolomics study.

Reporting for specific materials, systems and methods

We require information from authors about some types of materials, experimental systems and methods used in many studies. Here, indicate whether each material, system or method listed is relevant to your study. If you are not sure if a list item applies to your research, read the appropriate section before selecting a response.

Materials & experimental systems

n/a	Involved in the study	
[x]	[ ]	Antibodies
[ ]	[x]	Eukaryotic cell lines
[x]	[ ]	Palaeontology and archaeology
[x]	[ ]	Animals and other organisms
[x]	[ ]	Human research participants
[x]	[ ]	Clinical data
[x]	[ ]	Dual use research of concern

Methods

n/a	Involved in the study	
[x]	[ ]	ChIP-seq
[x]	[ ]	Flow cytometry
[x]	[ ]	MRI-based neuroimaging

Eukaryotic cell lines

Policy information about cell lines

[illegible]ell line source(s)	MDA-MB-231 NQO1(-), NQO1(+): giftofDr. David Boothman, Indiana University<br><br>SW620, SW480: ATCC<br>M059J, M059K: ATCC<br>SCC-61, rSCC-61: giftofDr. Cristina Furdui, Wake Forest University
Authentication	None of the cell lines were authenticated.
Mycoplasma contamination	All cell lines tested negative for Mycoplasma.
Commonly misidentified lines<br>(See ICLAC register)	None.
